# The treatment of a hypertrophic bleb after XEN gel implantation with the “Drainage Channel with sutures” method: a case report

**DOI:** 10.1186/s12886-019-1249-0

**Published:** 2019-12-03

**Authors:** Kamil Yavuzer, Ali Meşen

**Affiliations:** University of Health Sciences, Van Training and Research Hospital, Ophthalmology Clinic, Van, Turkey

**Keywords:** Glaucoma, Hypertrophic bleb, MIGS, Overfiltration, XEN gel implant

## Abstract

**Background:**

The placement of a XEN gel stent is an ab-interno, minimally invasive glaucoma surgery which provides a subconjunctival drainage pathway and decreases intraocular pressure (IOP).

**Case presentation:**

A 75-year-old male patient who had undergone XEN45 gel implantation after phacoemulsification and intraocular lens implantation appealed to the clinic. A filtration bleb was seen that extended through the nasal 180 degrees of the eye which caused ectropion of the lower eyelid. The value of the IOP was 12 mm Hg (mmHg). By the “Drainage Channel with Sutures” method this complication was effectively treated. As with every new method, there is a lack of knowledge about long-term outcomes in terms of effectiveness, technique and complications.

**Conclusion:**

The “Drainage Channel with Sutures” method has not been described in the literature yet. By this minimal invasive method, hypertrophic bleb complication of XEN gel implant has been successfully treated.

## Background

Glaucoma is a blinding optic neuropathy affecting nearly 60 million people world-wide. Among all forms of glaucoma, primary open angle glaucoma is the most frequent etiology [1]. The therapeutic arsenal for primary open angle glaucoma includes pressure lowering medications, laser treatments and surgery. Trabeculectomy and shunt surgery come with a range of complications like hypotony, leakage, shallowing of the anterior chamber and choroidal effusion as well as valve-related complications such as encapsulation, tube blockage, erosion and endothelial cell loss [2]. A novel technique of creating an alternative route through an ab interno approach via implantation of a collagen implant XEN has been described in an attempt to overcome the different complications which are seen in both trabeculectomy and shunt operations [3]. As with every new method, there is a lack of practitioner experience and knowledge about long-term outcomes in terms of effectiveness, technique and complications.

In this case report, we aimed to present a hypertrophic bleb complication after the third month of XEN gel implantation and to evaluate complication management. Similar studies have not yet been published. For that reason, this study contains novel methods and results. However, by this minimal invasive method, hypertrophic bleb complication of XEN gel implant has been successfully treated.

## Case presentation

A 75-year-old male patient who recently was accepted for a decrease in vision and who was treated for glaucoma appealed to the clinic. He had a longstanding history of pseudoexfoliation glaucoma which had been previously treated with topical medications.

His best corrected visual acuity was 6/38 (Snellen chart) in the right and left eyes. Intraocular pressure was 19 mmHg in the right eye and 23 mmHg in the left eye (Goldman applanation tonometry). Deep anterior chamber and presence of pseudoexfoliation materials with corticonuclear cataract was seen in both eyes by slit-lamp examination. Gonioscopic examination of both eyes has revealed open iridocorneal angle in all quadrants (Shaffer-Kanski classification system, Grade 4). On posterior segment examination we observed bilateral glaucomatous neuroretinal rim loss and vertical cup / disc ratio of each eye was 0.8.

Combinated cataract glaucoma surgery was planned for the patient, and XEN45 (Allergan, Irvine, California, USA) gel implantation was performed from the upper-nasal quadrant after phacoemulsification and intraocular lens implantation. After subconjunctival/sub-Tenon 0.1 ml (mL) lidocaine [20 mg (mg) / mL] injection to create an area for XEN implantation, 0.1 mL 0.2 mg / mL mitomycin C was applied to the implantation site in the upper nasal quadrant towards the posterior region with gentle massage. Stent placed to anterior trabecular meshwork as ab interno approach and by using intraoperative gonioscopy its placement was confirmed. At the end of surgery, the recommended placement of the XEN gel implant 1–2-3 mm (anterior chamber, sclera and subconjunctival/sub-Tenon’s area, respectively) was confirmed and surgery completed uneventfully.

**Fig. 1:**
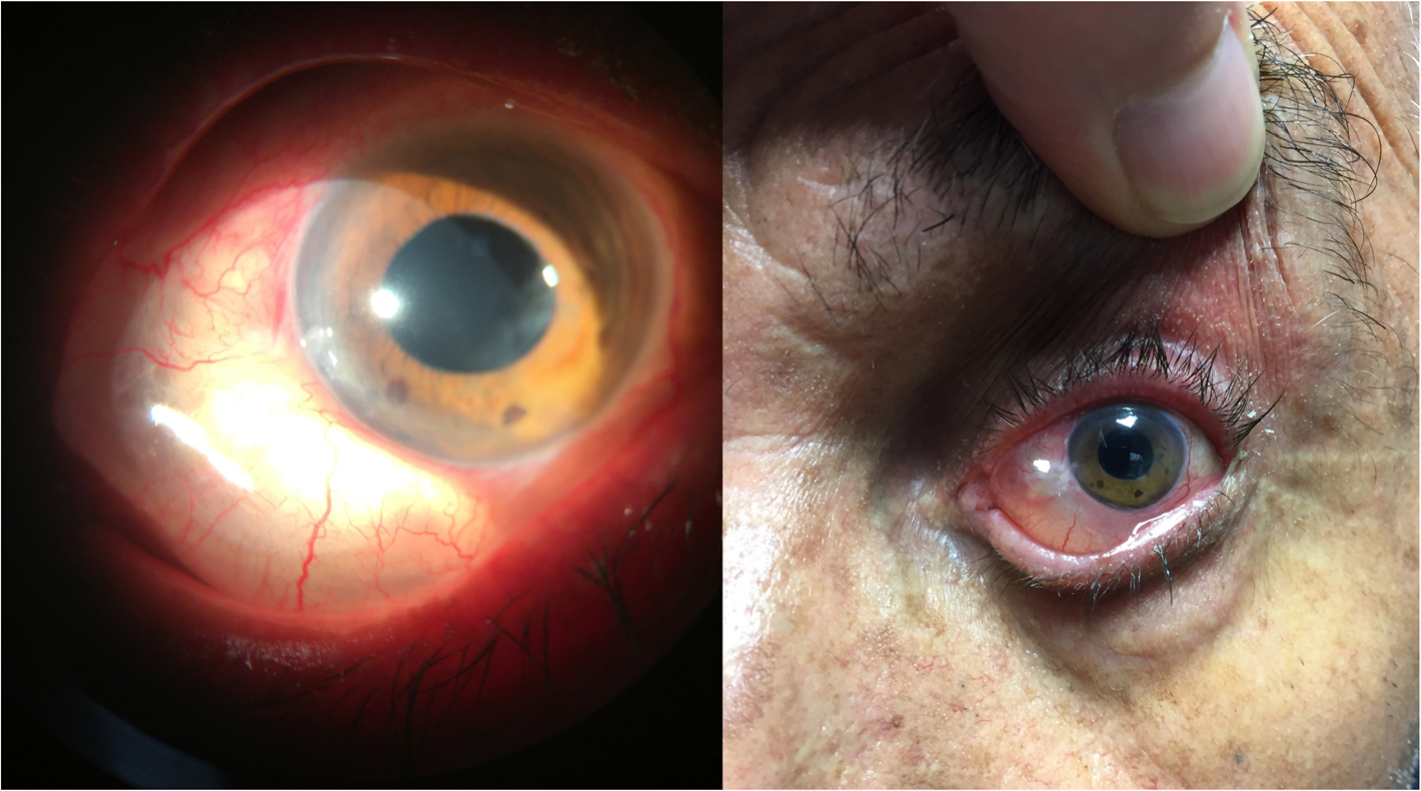
Hypertrophic filtration bleb caused ectropion of the lower eyelid

**Fig. 2:**
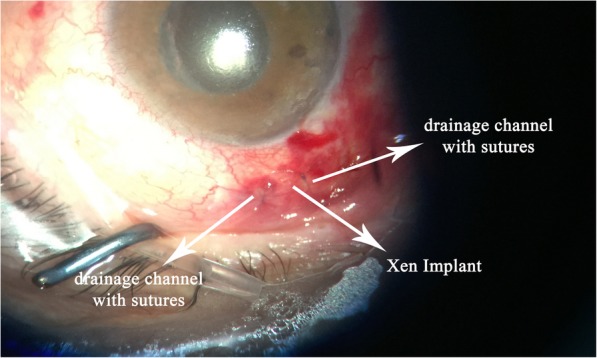
The “Drainage Channel with Sutures” extending from both sides of the XEN gel implant to the globe equator

**Fig. 3:**
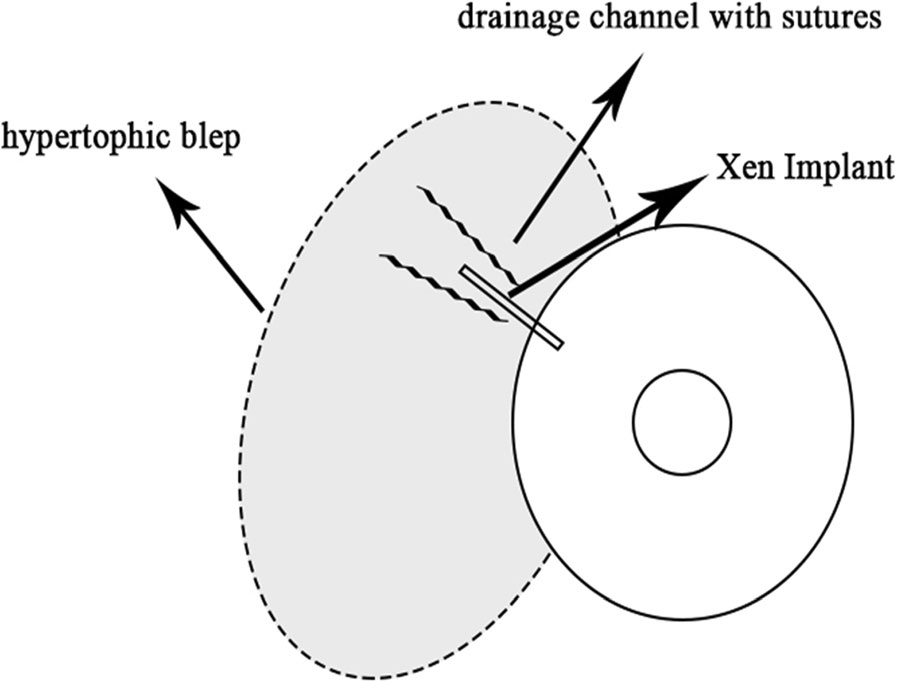
Schematic diagram of “Drainage Channel with Sutures” technique application

**Fig. 4:**
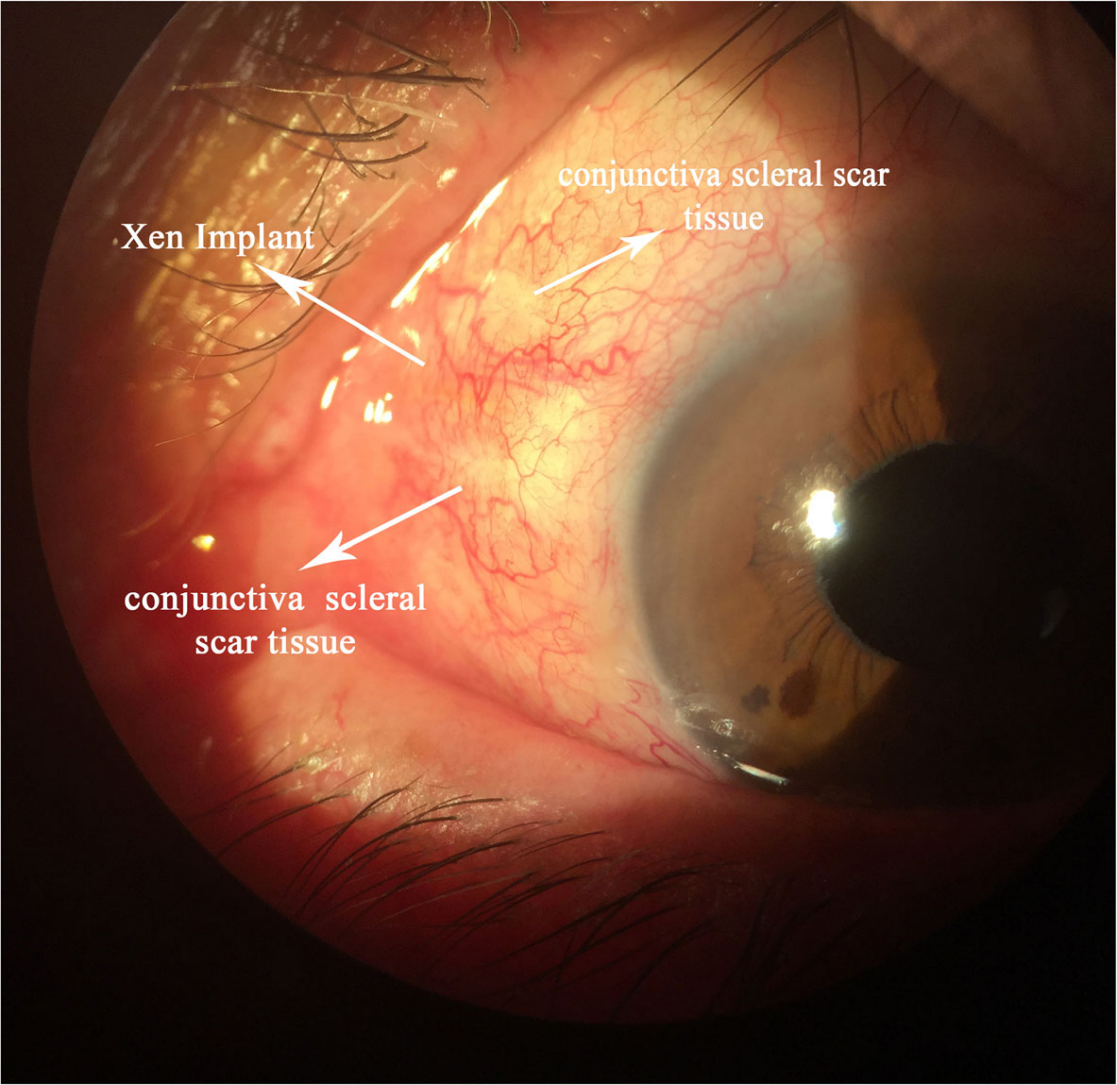
Image of XEN gel and the “Drainage Channel with Sutures” after l year

In the first 3 months, the intraocular pressure ranged from 9 to 13 mmHg without drug use, and no complications were determined. At the third month control, a bleb was seen that extended through the nasal 180 degrees of the eye which caused ectropion of the lower eyelid, and the value of the IOP was 12 mmHg (Fig. 1). Considering that the large bleb was linked to overfiltration, topical/systemic carbonic anhydrase treatment and tight closure were performed. Although the IOP was 7 mmHg after treatment, there was not any change in the size of the bleb. The clinic personnel thought this was a hypertrophic bleb, and the bleb was drilled by suture needle. In order to avoid a recurrence, a “Drainage Channel with Sutures,” extending from both sides of the XEN gel implant to the globe equator, was created (Fig. 2 and 3). This procedure was carried out with topical proparacain eye drops. A conjunctiva/scleral suture was made with 8/0 polyglactin to create scar tissue. While the nasal conjunctiva and the lower eyelid were observed quiet at the one-year follow-up appointment with the patient (Fig. 4), the IOP was measured at around 13 mmHg.

## Discussion and conclusion

XEN gel implant is promising glaucoma drainage surgery in terms of short operation time, rapid healing process, fewer complications and easy applicability. The implant utilizes subconjunctival filtration, creating a nonphysiologic route for aqueous outflow which is the basis of the traditional trabeculectomy and aqueous shunt glaucoma surgeries [2].

Several articles in the literatures have reported on relatively frequent complications such as hypotonia, choroidal effusion or choroidal folds, hyphema, the Seidel sign and a flat anterior chamber [4]. The filtration blebs normally produced are usually diffuse and not very large (approximately 3 h quadrant). In the present case, at the third month control, the patient presented a significant bleb that produced mechanical ectropion, a complication which is rarely described in the literature. When medical and conservative treatment do not resolve the situation, surgical treatment is indicated which comprises bleb resection and conjunctival stitches or autologous injection of platelet concentrates [5].

A similar case using fibrin adhesive has been previously described [6]. However, this method comprises the adhesion of all of the bleb and the likelihood of increased IOP. Therefore, instead of using fibrin adhesive, we preferred to use direct liquid drainage towards the back of the globe.

In conclusion, as with every new method, there is a lack of knowledge about long-term outcomes in terms of effectiveness, technique and complications. This case report shows that hypertrophic bleb complication of XEN gel implant can be successfully treated by “Drainage Channel with Sutures” method but stronger evidence is needed.

## Data Availability

Not applicable.

## Abbreviations

IOP: Intraocular pressure
mg: milligrams
MIGS: Minimally invasive glaucoma surgery
mL: milliliters
mmHg: millimeters of mercury
